# Aqueous Extract of *Solanum nigrum* Leaf Activates Autophagic Cell Death and Enhances Docetaxel-Induced Cytotoxicity in Human Endometrial Carcinoma Cells

**DOI:** 10.1155/2012/859185

**Published:** 2012-11-08

**Authors:** Cheng-Jeng Tai, Chien-Kai Wang, Yu-Jia Chang, Chi-Shian Lin, Chen-Jei Tai

**Affiliations:** ^1^Division of Hematology and Oncology, Department of Internal Medicine, Taipei Medical University Hospital, Taipei 110, Taiwan; ^2^Department of Internal Medicine, School of Medicine, College of Medicine, Taipei Medical University, Taipei 110, Taiwan; ^3^Department of Chinese Medicine, Taipei Medical University Hospital, 252 Wu Hsing Street, Taipei 110, Taiwan; ^4^Department of Obstetrics and Gynecology, School of Medicine, College of Medicine, Taipei Medical University, Taipei 110, Taiwan; ^5^Graduate Institute of Clinical Medicine, College of Medicine, Taipei Medical University, Taipei 110, Taiwan; ^6^Department of Surgery, Taipei Medical University Hospital, Taipei 110, Taiwan; ^7^Division of General Surgery, Department of Surgery, Taipei Medical University Hospital, Taipei Medical University, Taipei 110, Taiwan

## Abstract

Chemotherapy is the main approach in dealing with advanced and recurrent endometrial cancer. An effective complementary ingredient can be helpful in improving the clinical outcome. Aqueous extract of *Solanum nigrum* leaf (AE-SN) is a principal ingredient for treating cancer patients in traditional Chinese medicinal practice but lacks sufficient evidence to verify its tumor suppression efficacy. This study evaluated the antitumor effects of AE-SN and also assessed the synergistic effects of AE-SN with docetaxel On the human endometrial cancer cell lines, HEC1A, HEC1B, and KLE. The activation of apoptotic markers, caspase-3 and poly-ADP-ribose polymerase, and autophagic marker, microtubule-associated protein 1 light chain 3 A/B, wAS determined to clarify the cell death pathways responsible for AE-SN induced tumor cell death. Results indicated that AE-SN-treatment has significant cytotoxicity on the tested endometrial cancer cells with accumulation of LC3 A/B II and demonstrated a synergistic effect of AE-SN and docetaxel in HEC1A and HEC1B cells, but not KLE cells. In conclusion, AE-SN treatment was effective in suppressing endometrial cancer cells via the autophagic pathway and was also capable of enhancing the cytotoxicity of docetaxel in human endometrial cancer cells. Our results provide meaningful evidence for integrative cancer therapy in the future.

## 1. Introduction

Endometrial cancer is the most common cancer of the female genital tract in North America and Europe [1, 2]. Although most endometrial cancer cases are diagnosed at an early stage with a relatively good prognosis for the overall 5-year survival rate in 80% of patients, approximately 10% to 20% of patients are diagnosed in advanced stages with lower 5-year survival rates [3]. In advanced stage cases, there is still about a 50% recurrence rate after postoperative adjuvant therapy [4], with an extremely poor prognosis in 19% to 24% of the patients [5, 6]. In contrast to early stage cases, which are mainly treated by surgery, multimodality therapy is the mainstay of treatment for advanced and recurrent endometrial cancer cases, with radiation and chemotherapeutic regimens being frequently combined [7, 8]. Currently, combination chemotherapy such as doxorubicin/cisplatin is well accepted for treating endometrial cancer, whereas novel combination strategy such as taxanes/platinum combination is also being tested. Nomura and colleagues performed a phase II study to examine the clinical outcome of docetaxel/cisplatin, docetaxel/carboplatin, and paclitaxel/carboplatin in endometrial cancer cases and suggested that the taxane-platinum combination is efficacious in comparison to doxorubicin/cisplatin therapy, particularly docetaxel/cisplatin and paclitaxel/carboplatin [9]. However, the toxic effects of chemotherapeutic regimens are always a critical concern when establishing treatment strategies for endometrial cancer, particularly in elderly patients. Refined adjuvant chemotherapy with reduced systematic side effects and fair clinical outcomes can improve the management of endometrial cancer.

In order to relieve the systematic side effects and improve the efficacy of chemotherapy, the use of complementary and alternative medicine (CAM) has become very popular among cancer patients during the last few decades, particularly in western countries [10–12]. Based on the use of natural products and well-established theoretical approaches, traditional Chinese medicine (TCM) is not only widely accepted by people with a Chinese background, but is also considered a potential CAM approach for adjuvant cancer therapy [13–15]. TCM formulas such as TJ-41 (Bu-Zhong-Yi-Qi-Tang) and PHY906 (Huang-Qin-Tang) have been suggested to attenuate the adverse toxicity and potentiate the antitumor effects of chemo/radiotherapy, whereas several herbs such as *Curcuma longa*, *Panax ginseng*, and *Astragalus membranaceus* have shown antitumor effects in both preclinical and clinical evaluations [14]. These studies indicated that specific medical herbs and formulas from TCM may be helpful in improving current adjuvant cancer therapy. Furthermore, identification of more medicinal herbs capable of enhancing the clinical performance of current cancer therapy has become very attractive in recent years. 

In TCM practice, a therapeutic formula normally combines several medicinal herbs and is processed into an aqueous extract mixture. Typically, there is one major herb in this formula responsible for relieving the target symptom, and other medicinal herbs are added to enhance the therapeutic effects or reduce the side effects of the major herb. *Solanum nigrum* (SN) is a medicinal herb widely used as an elemental ingredient in TCM formulas for TCM cancer therapy [16]. In recent years, the *in vitro* anti-tumor effects of SN extracts have been observed in various cancer types, such as leukemia, prostate, liver, breast, lung, stomach, colon, and bladder cancers [16–18]. In a TCM formula, the SN ingredient is prepared through aqueous extraction of the leaf part. Two pioneer studies verified that the SN-related anti-tumor effects in human breast cancer and hepatocarcinoma cells were due to activation of apoptotic and autophagic cell death using aqueous extract of SN (AE-SN) [17, 19]. These results suggested that AE-SN may serve as an adjuvant ingredient capable of further enhancing the tumor suppression efficiency of chemotherapeutic regimens. 

Currently there is no evidence to verify the synergistic or additive effects of AE-SN on standard chemotherapeutic regimens in human cancer cells. Since AE-SN activates apoptotic and autophagic cell death in cancer cells, it may achieve a synergistic or additive effect with chemotherapeutic regimens which induce cancer cell death in other pathways. For example, the anti-tumor efficacy of docetaxel, which is mainly achieved by stabilizing microtubules followed by cell cycle arrest and caspase-associated cell apoptosis [20, 21], may be further enhanced by cotreatment with AE-SN. The aim of the present study therefore is first to examine the antitumor effects of AE-SN in human endometrial cancer cells and also clarify whether AE-SN is capable of further enhancing the antitumor effects of docetaxel in human endometrial cancer cells or not.

## 2. Materials and Methods

### 2.1. Plant Materials and Preparation of AE-SN

In TCM practice, medicine formulas which are prepared as aqueous extracts are generally given to patients orally. The use of the SN leaf in TCM cancer therapy also follows this principle. In order to evaluate the anti-tumor effects of the SN leaf, the preparation of AE-SN used in this study was based on the TCM processing method. Briefly, 50 g of the dried leaf part of *Solanum nigrum* was immersed in 750 mL of distilled water. This raw solution was then slowly heated to 100°C within 50 min, followed by another 1 hr at 100°C. The AE-SN was further condensed into a final concentration of 1 g/mL at 100°C and used for the following experiments.

### 2.2. Cell Culture

The human endometrial carcinoma cell lines, HEC1A, HEC1B, and KLE, were purchased from American Type Culture Collection (ATCC, Manassas, VA, USA). HEC1A and 1B are moderately differentiated, estrogen-dependent adenocarcinoma cells, whereas KLEs are poorly differentiated, estrogen-independent adenocarcinoma cells. Cells were cultured in Dulbecco's modified Eagle medium/nutrient mixture F-12 medium (Gibco, Grand Island, NY, USA) with 100 U/mL of penicillin and 100 *μ*g/mL streptomycin (Invitrogen Life Technologies, Carlsbad, CA, USA) at 37°C in a 5% CO_2_ humidified incubator.

### 2.3. Cell Viability Assay and Microscopic Observation

Cells were seeded into 96-well microplates at a density of 5 × 10^3^ cells per well for HEC1A and HEC1B cells and 1 × 10^4^ per well for KLE cells. After overnight incubation, cells were then treated with 0, 0.05, 0.1, 0.2, 0.5, 1, 2, and 5 mg/mL AE-SN for 24 or 48 hr to determine the cytotoxicity of AE-SN. In the synergistic study of docetaxel and AE-SN, cells were treated with 0 to 100 nM docetaxel with 0, 0.2, and 0.5 mg/mL AE-SN for 48 hr. Cell morphology was observed with a Nikon Eclipse TS100 optical microscope (Nikon Instruments, Melville, NY, USA) and photographed at 100x magnification. The cell viability was determined by 3-(4, 5-Dimethylthiazol-2-yl)-2, 5-diphenyltetrazolium bromide (MTT) assay. An addition of 30 *μ*L 5 mg/mL MTT phosphate buffered saline solution (Bio Basic Inc, Markham, Ontario, Canada) was made to each well, followed by incubation at 37°C for 3 hr. After incubation, the culture medium containing the MTT solution was replaced by 100 *μ*L dimethyl sulfoxide (JT Baker, Phillipsburg, NJ, USA) and shaken in the dark for 15 min at room temperature for complete dissolution of the MTT formazan productions. The optical density of each well was measured by absorbance at 550 nm using an Emax microplate reader (Molecular Devices, Sunnyvale, CA, USA). The half maximal inhibitory concentration (IC_50_) of AE-SN was determined by MTT assay results using CalcuSyn software (Biosoft, Cambridge, UK). 

In TCM practice, AE-SN is normally prepared in a range of 11.25 to 37.5 g raw material and given to patients by daily oral administration. A presumed conversion of the oral dose of AE-SN and the *in vitro* treatment concentration is based on the administrated raw material weight and the volume of total body fluid. For example, 37.5 g of SN raw material for preparing AE-SN is given to an adult male with an assumed total body-fluid volume of 45 L. Accordingly, the final concentration of AE-SN after absorption is approximately 0.83 mg/mL for this adult male. When the IC_50_ of AE-SN on tested endometrial cancer cells is lower than 0.83 mg/mL, the antitumor effect of AE-SN is considered effective for oral administration. 

A trypan blue exclusion test was also performed to confirm the cell viability determined by MTT assay. Cells were seeded into 24-well plates at a density of 3 × 10^4^ cells per well for HEC1A and HEC1B cells and 6 × 10^4^ cells per well for KLE cells. After overnight incubation, cells were treated with 0.1, 0.5, or 2 mg/mL AE-SN and incubated for the next 48 hr. Cells were then harvested by trypsinization and centrifuged at 100 ×g for 5 min. Cell pellets were suspended in prewarmed phosphate buffered saline and mixed with trypan blue (Sigma-Aldrich Corp, St Louis, MO, USA) in a 1 : 1 ratio for 3 min at room temperature. The number of live cells without trypan blue staining was counted using a hemocytometer under a microscope by two independent observers.

### 2.4. Western Blotting Analysis of Cell Death Markers

 HEC1A, HEC1B (5 × 10^5^ cells per dish), and KLE (1 × 10^6^ per dish) cells were seeded in 6 cm dishes overnight and incubated with 0, 0.5, and 1.0 mg/mL AE-SN for the next 24 or 48 hr. Cells were harvested by RIPA buffer (150 mM NaCL, 50 mM pH 7.5 Tris-HCL, 1% NP-40, 0.5% deoxycholate, 0.1% SDS, 1 mM PMSF, 10 *μ*g/mL leupeptin, and 100 *μ*g/mL aprotinin). The total protein concentration of the cell extracts was determined by a Bio-Rad protein assay kit (Bio-Rad Laboratories, Hercules, CA, USA). Each cell extract was then equalized to 30 *μ*g and separated using 12% sodium dodecyl sulfate polyacrylamide gel electrophoresis. The proteins were transferred into a polyvinylidene fluoride membrane (Pall Corp, Port Washington, NY, USA) and probed with the primary antibodies, poly-ADP-ribose polymerase, (PARP, 1 : 1,000), caspase-3 (1 : 1,000), mammalian microtubule-associated protein 1 light chain 3 A/B (LC3A/B, 1 : 1,000), and glyceraldehyde 3-phosphate dehydrogenase (GAPDH, 1 : 10,000), followed by donkey anti-rabbit horseradish peroxidase conjugated secondary antibody (1 : 10,000, Santa Cruz Biotechnology, Santa Cruz, CA, USA). Anti-PARP, caspase-3, and LC3 A/B were purchased from Cell Signaling Technology (Danvers, MA, USA), and anti-GAPDH was purchased from Abfroniter (Seoul, Korea). Immunoreactivity was then detected with a WesternBright electrochemiluminescence western blotting detection kit (Advabsta, Menlo Park, CA, USA). Semiquantitative analysis of the intensity of immunoreactive bands was performed by ImageJ software (National Institutes of Health, Bethesda, MD, USA).

### 2.5. Microscopic Observation and Immunofluorescence Staining of LC-3 A/B

HEC1A, HEC1B (5 × 10^5^ cells per dish), and KLE (1 × 10^6^ per dish) cells were seeded on chamber slides overnight and then incubated with 0 or 1.0 mg/mL AE-SN for the next 48 hr. Cells were then fixed in 3% formaldehyde and permeabilized by ice-cold methanol. Localization of LC3A/B was performed by incubation of LC3 A/B primary antibody in 1 : 100 dilution, followed by Alexa Fluor 488-goat anti-rabbit secondary antibody (1 : 200, Invitrogen Life Technologies, Carlsbad, CA, USA). Counterstaining of cell nuclei was performed by Vectashield H-1200 mounting medium (Vector Laboratories, Burlingame, CA, USA). Fluorescent staining was examined with an Olympus IX81 fluorescence microscope (Olympus, Melville, NY, USA) and photographed by a Hamamatsu digital camera (Hamamatsu, HamamatsuCity, Japan).

### 2.6. Statistical Analysis

Data from cell viability and semiquantitative western blotting analysis were presented as mean ± standard derivation (SD). Statistical significance was analyzed by Student's *t*-test in comparing two different groups and analyzed by one-way ANOVA in examination of the dose-dependent effect. Statistical analysis was performed by SPSS (SPSS Inc, Chicago, IL, USA).

CalcuSyn software (Biosoft, Cambridge, UK) was used for the statistical analysis of the synergistic effects of combined treatment with docetaxel and AE-SN. Statistical analysis of the synergic effect with the CalcuSyn software is based on the median-effect method and evaluates drug-combination effects with the combination index (CI) value. When the CI value is less than 1, the drug-combination effect is considered a synergistic effect [22].

## 3. Results

### 3.1. Cytotoxicity of AE-SN in Human Endometrial Adenocarcinoma HEC1A, HEC1B, and KLE Cells

**Figure 1:**
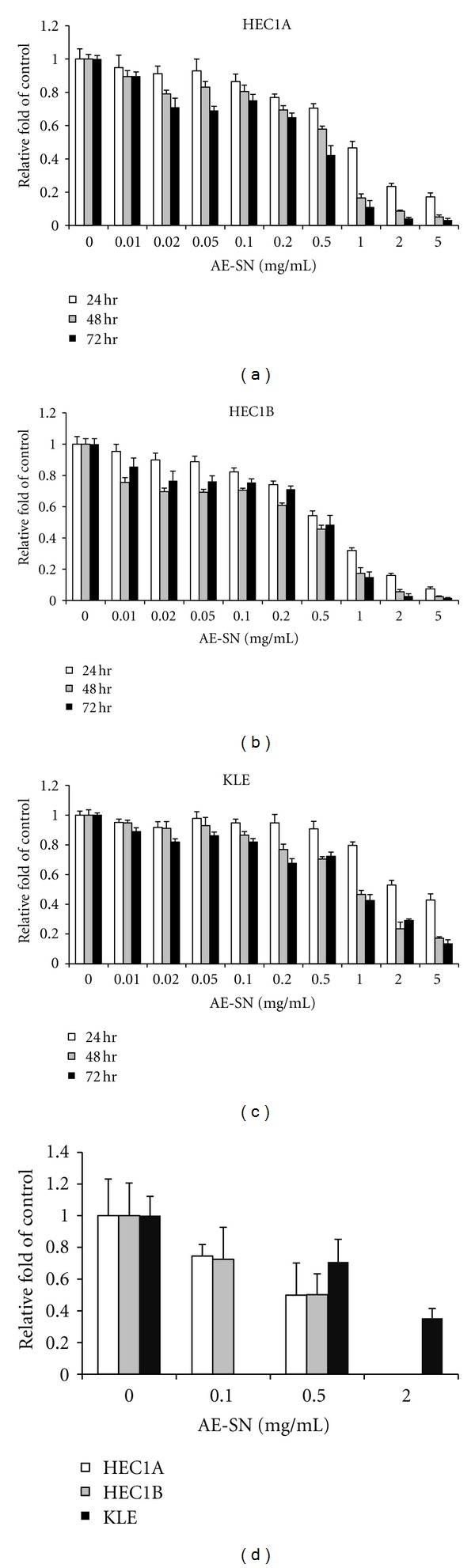
Cytotoxicity of AE-SN in human endometrial cancer cells. (a–c) Results of MTT assay in HEC1A, HEC1B, and KLE cells. Cells were treated with 0, 0.05, 0.1, 0.2, 0.5, 1, 2 or 5 mg/mL AE-SN for 24, 48, or 72 hr. All three cell lines showed dose-dependent effects with AE-SN treatment at the three time points, as shown by one way ANOVA (*P* < 0.001). (d) Trypan blue exclusion test in HEC1A, HEC1B, and KLE cells. HEC1A and HEC1B cells were treated with 0, 0.1 or 0.5 mg/mL AE-SN, whereas KLE cells were treated with 0.5 and 2 mg/mL AE-SN for 48 hr. All three cell lines showed dose-dependent effects with AE-SN treatment, as shown by one-way ANOVA (*P* = 0.042, 0.043, and 0.001 for HEC1A, HEC1B, and KLE cells, resp.). Experiments were done in triplicate, and all data are presented as mean ± SD.

**Figure 2:**
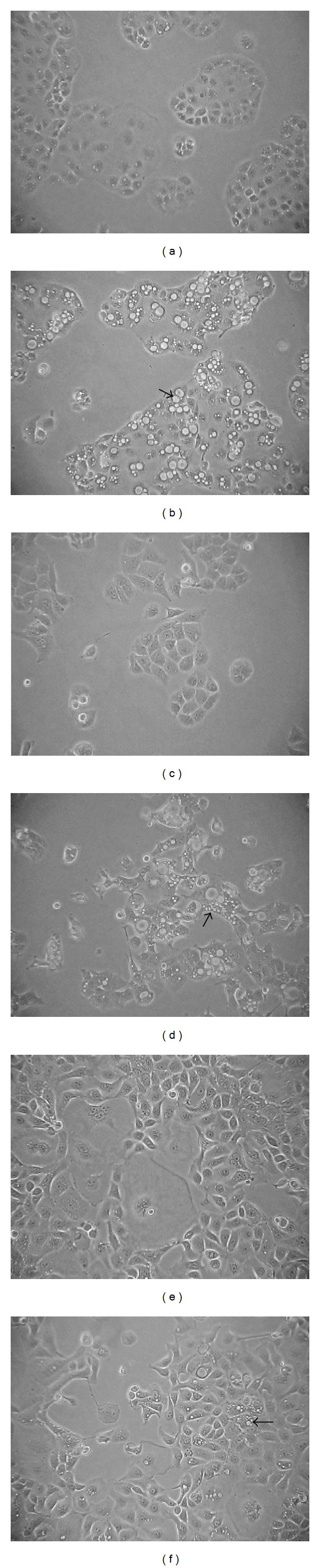
Morphological changes in AE-SN-treated human endometrial cancer cells. ((a) and (b)) HEC1A cells were treated with 0 or 0.5 mg/mL AE-SN for 48 hr. ((c) and (d)) HEC1B cells were treated with 0 or 0.5 mg/mL AE-SN for 48 hr. ((e) and (f)) KLE cells were treated with 0 or 0.5 mg/mL for 48 hr. Arrows indicate lipid droplet-like morphological changes in AE-SN-treated cells. (100x magnification).

 To evaluate whether AE-SN reduces cell viability in HEC1A, HEC1B, and KLE cells, a gradient concentration of AE-SN (0 to 5 mg/mL) and the incubation time (24, 48, and 72 hr) were tested in the present study. As shown in Figure 1, MTT assay results indicated that treatment with AE-SN-reduced cell viability in a dose-dependent manner in all three human endometrial adenocarcinoma cell lines. These MTT assay results were further confirmed by a trypan blue exclusion test (Figure 1(d)). Since the AE-SN showed gradient cytotoxic effects in all three cell lines, the IC_50_ could also be determined using the computer software, CalcuSyn. Considering that the cell viability at 72 hr incubation may be affected by cell starvation, the cell viability at 48 hr incubation was chosen for IC_50_ analysis. The analyzed results suggested that the IC_50_ of AE-SN was 0.56 mg/mL in HEC1A, 0.38 mg/mL in HEC1B, and 1.60 mg/mL in KLE cells. AE-SN-treated cells also demonstrated lipid droplet-like morphological changes, particularly with HEC1A and HEC1B cells (Figure 2). In contrast, no apoptotic-related morphological changes such as cell shrinkage or chromatin condensation were observed in AE-SN-treated cells. The results of cell viability and morphological changes suggested that compared with that in KLE cells, AE-SN treatment was more effective in HEC1A and HEC1B cells. The IC_50_ values of HEC1A and HEC1B were lower than 0.83 mg/mL and presumably could be effective for tumor suppression in comparison with oral administration of TCM practice.

### 3.2. Determination of Cell Death-Related Protein Markers in AE-SN-Treated Endometrial Carcinoma Cells

**Figure 3:**
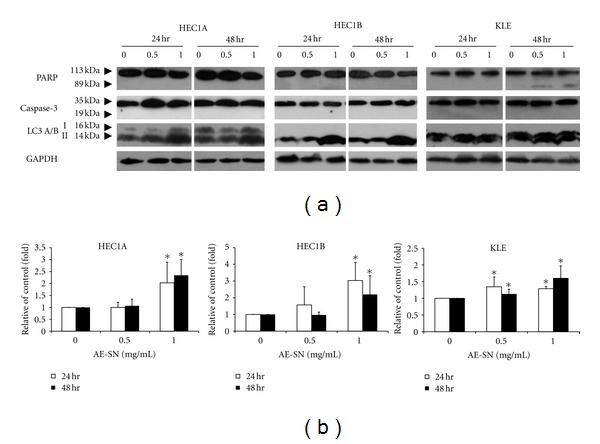
Detection of cell death markers in AE-SN-treated human endometrial cancer cells. (a) HEC1A, HEC1B and KLE cells were treated with 0, 0.5, and 1.0 mg/mL AE-SN for 48 hr, and total protein extracts were harvested for western blotting analysis of the cell death markers, PARP, caspase-3 and LC3 A/B. (b) Accumulation of LC3 A/B II was semiquantified by Image J software. All experiments were replicated five times for statistical analysis of semiquantified data and are presented as mean ± SD. *Indicates statistical significance compared with 0 mg/mL AE-SN treatment by Student's *t*-test (*P* < 0.05).

**Figure 4:**
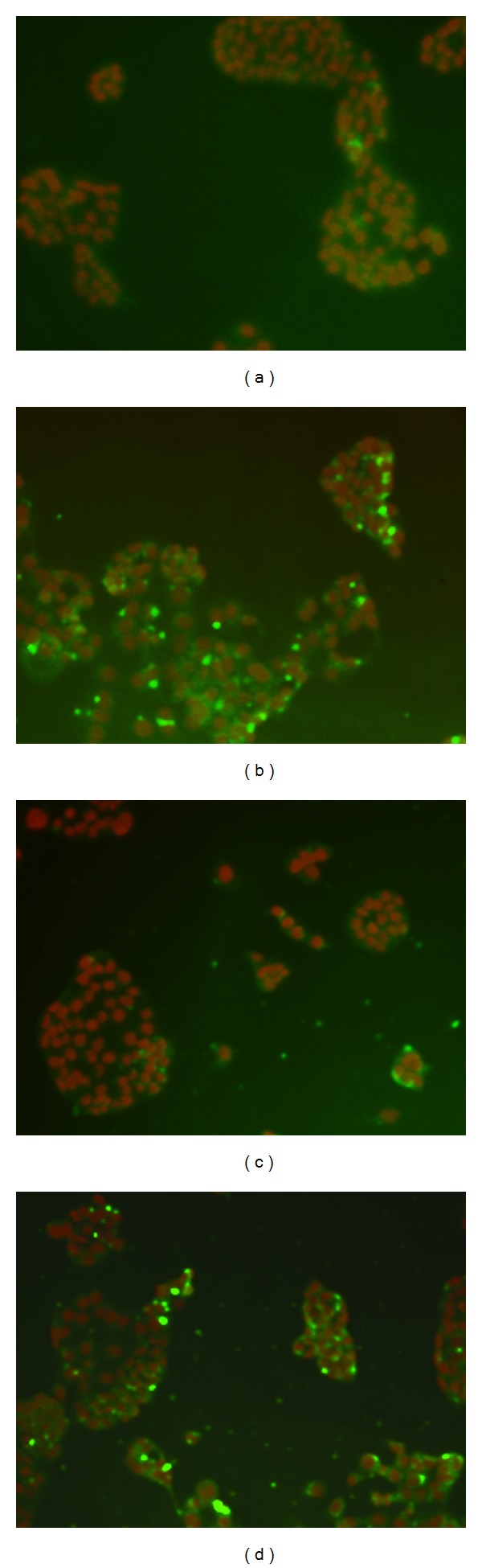
Localization of LC3 A/B accumulation in AE-SN-treated HEC1A and HEC1B cells. ((a) and (b)) HEC1A cells were treated with 0 or 0.5 mg/mL AE-SN for 48 hr. ((c) and (d)) HEC1B cells were treated with 0 or 0.5 mg/mL AE-SN for 48 hr. Red, pseudocolorization on DAPI-stained cell nuclei. Green, LC3 A/B-stained cytoplasm (100x magnification).

To clarify whether AE-SN treatment reduced cell viability by activating programmed cell death in HEC1A, HEC1B, and KLE cells, activation of three principle cell death markers, PARP, caspase-3, and LC-3 A/B, was determined by western blotting analysis. Cleaved caspase-3 is a cell death marker for caspase-dependent apoptosis, whereas PARP is a cell marker activated during both apoptosis and necrosis by cleavage. On the other hand, accumulation of LC-3 A/B-II indicates the activation of autophagic cell death. Results shown in Figure 3(a) indicated no cleavage of PARP and caspase-3 when all three cell lines were treated with AE-SN, whereas the accumulation of LC-3 A/B-II was statistically significant in all three cell lines (Figure 3(b)). Furthermore, the activation of LC-3 A/B-II was observed in the cytoplasm in AE-SN-treated HEC1A and HEC1B cells as shown by immunofluorescent staining (Figure 4). Together, these results suggested that AE-SN mainly activated autophagic rather than apoptotic and necrotic cell death in the three tested human endometrial adenocarcinoma cell lines.

### 3.3. Synergistic Effects of AE-SN in Docetaxel-Treated HEC1A and HEC1B Cells

**Figure 5:**
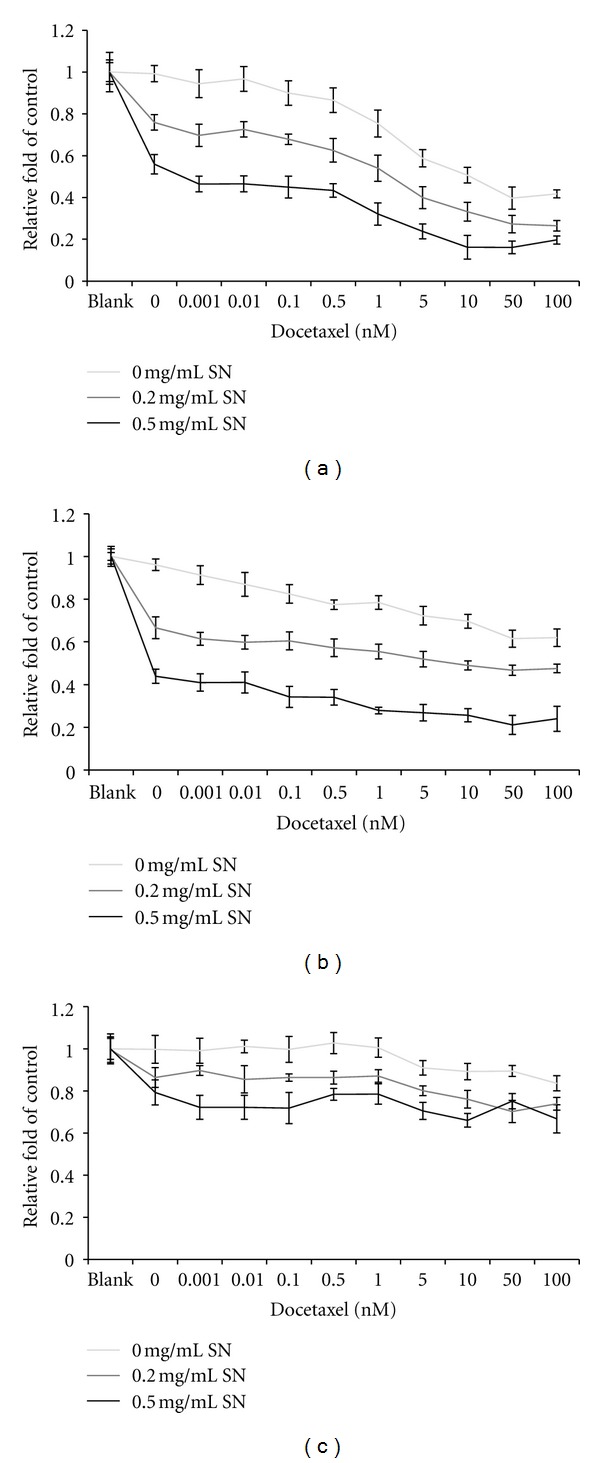
Cytotoxicity of AE-SN and docetaxel cotreatment in human endometrial cancer cells. (a) HEC1A cells. (b) HEC1B cells. (c) KLE cells. Tested cells were treated with serial doses of docetaxel from 0 to 100 nM together with 0, 0.2, or 0.5 mg/mL AE-SN for 48 hr. Cell cytotoxicity was determined by MTT assay and presented as mean ± SD. Experiments were performed in triplicate.

Since previous results verified that AE-SN treatment resulted in significant cell cytotoxicity in HEC1A, HEC1B, and KLE cells and was associated with autophagic cell death, the next step was to clarify the combination effect of AE-SN and a chemotherapeutic regimen with docetaxel in human endometrial adenocarcinoma cells. According to the IC_50_ determined by the cell viability study, doses of 0.2 and 0.5 mg/mL AE-SN which covered the IC_50_ dosage were chosen for cells treated with 0 to 100 nM docetaxel. The results shown in Figure 5 demonstrated that a combination effect was observed in HEC1A and HEC1B cells, whereas KLE cells were not only less sensitive to AE-SN, but also resistant to docetaxel treatment. The CI was calculated for AE-SN and docetaxel-treated cells and showed that a synergistic effect occurred in both 0.2 and 0.5 mg/mL AE-SN-treated HEC1A and HEC1B cells treated with 1 to 100 nM docetaxel (Table 1). These results suggested that cotreatment with AE-SN further enhanced the cell cytotoxicity of docetaxel in HEC1A and HEC1B cells.

## 4. Discussion

The leaf part of SN is one of the principle components in many TCM formulas used to treat cancer patients, but the antitumor efficacy and biological mechanism of the SN leaf have not been investigated until very recently. A pioneer study published in 2010 demonstrated that the aqueous extract of SN leaf induced both apoptotic and autophagic cell death on human hepatocellular and breast carcinoma cells [17]. Another study suggested that the aqueous extract of SN leaf suppressed melanoma growth and invasion both* in vivo* and *in vitro* [23]. In the present study, the aqueous extract of SN leaf also showed a cytotoxic effect in human endometrial cancer cells, and the antitumor efficiency was dependent on the cell type. HEC1A and HEC1B cells isolated from well-differentiated endometrial adenocarcinoma are more sensitive to AE-SN treatment (IC_50_ = 0.38 and 0.56 mg/mL, resp.) than KLE cells that are isolated from poorly-differentiated endometrial adenocarcinoma (IC_50_ = 1.60 mg/mL). Moreover, HEC1A and HEC1B cells are considered estrogen-dependent carcinoma cells [24], whereas KLE cells are estrogen-independent [25]. Among the tested endometrial adenocarcinoma cells, the accumulation of the autophagic cell marker, LC-3 A/B II, was at a relatively lower level in KLE cells than HEC1A and HEC1B cells. This result may explain why KLE cells were less sensitive to AE-SN treatment. We speculated that the poor differentiation and estrogen-independent features of KLE cells may be related to AE-SN resistance, but more evidences are required for further clarification.

Interestingly, the aqueous extract of SN leaf activated both apoptosis and autophagic cell death in human breast adenocarcinoma AU565 cells [17], but only activated autophagic cell death in human endometrial adenocarcinoma HEC1A, HEC and KLE cells. Cell markers such as PARP and caspase-3 were not cleaved to active forms in AE-SN treated-endometrial adenocarcinoma cells, suggesting that the tested HEC1A, HEC1B, and KLE cells were capable of evading the apoptotic pathways activated by AE-SN, but were still vulnerable to AE-SN-induced autophagic cell death. However, currently the effective compounds responsible for AE-SN-induced tumor cell death are not clear. Although Huang and colleagues suggested that the flavonoids present in AE-SN are the major compounds which induce cell death in human breast adenocarcinoma AU565 cells [17], it is not clear whether flavonoids activate apoptosis, autophagic cell death, or both. Other studies suggested that solamargine isolated from SN is the key compound inducing tumor cell death. However solamargine only activated caspase-3-dependent apoptosis in human leukemia and hepatocellular carcinoma cells [26, 27]. The specific effective compounds in SN leaf which activate apoptosis and autophagic cell death would necessitate further clarification. These pioneer studies together suggest that AE-SN induced tumor cell death may not rely on a single effective compound but is synergized by several functional compounds to activate multiple cell death pathways, including apoptosis and autophagy, in tumor cells. Furthermore, the activated cell death pathways are dependent on the specific tumor cell type. For example AE-SN only activated autophagic cell death in the human endometrial cancer cell lines, HEC1A, HEC1B, and KLE, but not apoptosis. 

Since chemotherapy is one of the main approaches to deal with advanced and recurrent endometrial cancer, the present study also evaluated the complementary role of AE-SN with a current chemotherapeutic regimen. In the present study, cotreatment of AE-SN and docetaxel significantly increased the cytotoxicity in HEC1A and HEC1B cells. However, docetaxel showed a weak cytotoxicity effect in KLE cells and cotreatment with AE-SN also failed to enhance the cytotoxicity. Although the biological mechanism of docetaxel-induced tumor cell death is not fully understood, current knowledge indicates that docetaxel first causes the stabilization of microtubulins by binding to beta-tubulins, thereby resulting in the activation of caspase-dependent apoptosis with consequent cleavage of PARP via mitochondrial pathways in lung, prostate, and ovarian cancers and melanoma cells [28–31]. Our data also confirmed that docetaxel treatment in human endometrial adenocarcinoma cells resulted in cleavage of PARP (data not shown). The AE-SN-activated autophagic pathway is therefore complementary to docetaxel-induced apoptotic cell death in tumor cells, and further enhances the overall cytotoxicity in endometrial cancer cells. Moreover, HEC1A cells are considered cisplatin-resistant cancer cells by protein kinase B activity [28, 32], and the cytotoxic effect of docetaxel and AE-SN cotreatment observed in HEC1A cells suggested the potential application of AE-SN and docetaxel in endometrial cancer cases with cisplatin-resistant features. 

In TCM practice, AE-SN is not only used for dealing with cancer, but also for treating inflammation, edema, and mastitis [33]. In the present study, human endometrial cancer cells were treated by AE-SN directly, and therefore the systematic effects of AE-SN are still unclear. Further study is required to clarify the systematic effects of AE-SN, particularly the unexpected side effects *in vivo*. In addition, although the IC_50_ of AE-SN in HEC1A and HEC1B cells suggested that the daily oral dose of AE-SN generally given in TCM practice is presumably reachable and effective in dealing with human endometrial cancer cells, the real absorption rate of AE-SN via the gastrointestinal tract and pharmacokinetic dosing remains undetermined. The optimal dosage of AE-SN to give alone or to accompany chemotherapeutic regimens such as docetaxel by oral administration therefore would need to be further clarified with *in vivo* studies. 

## 5. Conclusion

**Figure 6:**
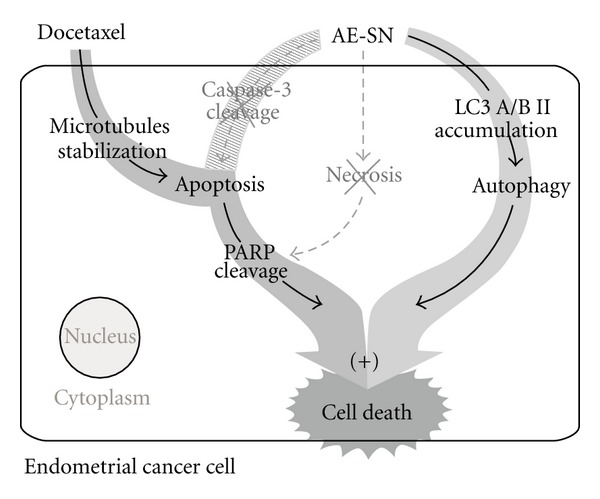
Proposed mechanism for the programmed cell death activated by AE-SN and docetaxel in human endometrial cancer cells. + indicates synergistic effect of AE-SN and docetaxel.

The aqueous extract of SN leaf, a widely used medicinal herb in TCM practice, demonstrated significant cytotoxicity in human endometrial cancer cells via activation of autophagic cell death. Furthermore, it was also capable of enhancing the tumor suppression efficacy of docetaxel in the human endometrial cancer cell lines, HEC1A and HEC1B (the proposed mechanism is shown in Figure 6). These *in vitro* results therefore suggest that the use of AE-SN could be potentially beneficial in dealing with endometrial cancer cells, particularly in a complementary role with chemotherapeutic drug such as docetaxel and may be of interest for further studies in developing integrative cancer therapy against endometrial cancer.

## Figures and Tables

**Table 1:** Synergistic effect of AE-SN and docetaxel on human endometrial carcinoma cells HEC1A and HEC1B. By analyzing the cytotoxicity on AE-SN and docetaxel co-treated HEC1A and HEC1B cells, the combination index (CI) values were obtained. When the CI value is less than 1, the effect is considered synergistic.

Docetaxel (nM)	AE-SN (mg/mL)			
	CI for HEC-1A		CI for HEC-1B	
	0.2	0.5	0.2	0.5
0.001	16.7	55.36	33.08	162.49
0.01	35.25	42.36	15.78	162.49
0.1	10.41	42.37	15.8	4.99
0.5	3.43	24.73	1.72	4.99
1	0.67	1.08	0.43	0.19
5	0.08	0.08	0.06	0.11
10	0.06	0.01	0.01	0.03
50	0.11	0.02	0.02	0.01
100	0.19	0.07	0.04	0.02
